# Investigating the Impact of Automation on the Health Care Workforce Through Autonomous Telemedicine in the Cataract Pathway: Protocol for a Multicenter Study

**DOI:** 10.2196/49374

**Published:** 2023-12-05

**Authors:** Sarah Khavandi, Fatema Zaghloul, Aisling Higham, Ernest Lim, Nick de Pennington, Leo Anthony Celi

**Affiliations:** 1 Ufonia Oxford United Kingdom; 2 Gloucestershire Hospitals NHS Foundation Trust Cheltenham United Kingdom; 3 Imperial College School of Medicine Imperial College London London United Kingdom; 4 Operations and Management Science Healthcare and Innovation University of Bristol Bristol United Kingdom; 5 Royal Berkshire NHS Foundation Trust Reading United Kingdom; 6 Department of Computer Science University of York York United Kingdom; 7 Laboratory for Computational Physiology Massachusetts Institute of Technology Cambridge, MA United States; 8 Division of Pulmonary, Critical Care and Sleep Medicine Beth Israel Deaconess Medical Center Boston, MA United States; 9 Department of Biostatistics Harvard TH Chan School of Public Health Boston, MA United States

**Keywords:** artificial intelligence, autonomous telemedicine, clinician burnout, clinician wellbeing, conversational agent, digital health, health communication, health information technology, health services, healthcare, medical informatics, socio-technical system approach, systems approach, technology acceptability

## Abstract

**Background:**

While digital health innovations are increasingly being adopted by health care organizations, implementation is often carried out without considering the impacts on frontline staff who will be using the technology and who will be affected by its introduction. The enthusiasm surrounding the use of artificial intelligence (AI)–enabled digital solutions in health care is tempered by uncertainty around how it will change the working lives and practices of health care professionals. Digital enablement can be viewed as facilitating enhanced effectiveness and efficiency by improving services and automating cognitive labor, yet the implementation of such AI technology comes with challenges related to changes in work practices brought by automation. This research explores staff experiences before and after care pathway automation with an autonomous clinical conversational assistant, Dora (Ufonia Ltd), that is able to automate routine clinical conversations.

**Objective:**

The primary objective is to examine the impact of AI-enabled automation on clinicians, allied health professionals, and administrators who provide or facilitate health care to patients in high-volume, low-complexity care pathways. In the process of transforming care pathways through automation of routine tasks, staff will increasingly “work at the top of their license.” The impact of this fundamental change on the professional identity, well-being, and work practices of the individual is poorly understood at present.

**Methods:**

We will adopt a multiple case study approach, combining qualitative and quantitative data collection methods, over 2 distinct phases, namely phase A (preimplementation) and phase B (postimplementation).

**Results:**

The analysis is expected to reveal the interrelationship between Dora and those affected by its introduction. This will reveal how tasks and responsibilities have changed or shifted, current tensions and contradictions, ways of working, and challenges, benefits, and opportunities as perceived by those on the frontlines of the health care system. The findings will enable a better understanding of the resistance or susceptibility of different stakeholders within the health care workforce and encourage managerial awareness of differing needs, demands, and uncertainties.

**Conclusions:**

The implementation of AI in the health care sector, as well as the body of research on this topic, remain in their infancy. The project’s key contribution will be to understand the impact of AI-enabled automation on the health care workforce and their work practices.

**International Registered Report Identifier (IRRID):**

PRR1-10.2196/49374

## Introduction

### Background and Rationale

There has recently been a rise in the use of artificial intelligence (AI) and automated technologies within health care that come with the promise of increasing efficiency and improving clinical workloads [1]. These AI technologies are already being shown to perform tasks previously entirely within the scope of human clinicians, from reading scans to replacing entire clinical consultations [2-4]. Despite the potential for AI to result in organizationally valuable outcomes, research shows that this is not always the case [5]. Many organizations invest effort, resources, and time into emerging technologies yet do not experience desired results, ultimately deeming these initiatives unsuccessful. It is evident that for AI implementation to be successful, the interrelationship between individuals, technologies, and the system should be observed holistically. It is important to take this holistic view into account because, as with the industrial revolution or more recent waves of digitization, workers who experience their role directly impacted by automation or digitization can either be empowered to “work at the top of their license” or feel even further disempowered and left behind.

Investigating the impact of such technologies on individuals is paramount in the current context of the global health care workforce crisis. Clinicians’ well-being is at risk, with studies to date showing that several factors can contribute to poor acceptance and adoption, leading to a negative impact on workloads and thus well-being. There are many definitions of well-being, and in this study, we define the term, in the context of the workplace (based on the Stanford Professional Fulfillment model) [6], as “the degree of intrinsic positive reward we derive from our work, including happiness, meaningfulness, contribution, self-worth, satisfaction, and feeling in control when dealing with difficult problems at work.”

This has great implications given the burnout epidemic and is becoming increasingly important to address after the recent COVID-19 pandemic, which took an unprecedented toll on an already pressurized system [7-9]. Burnout has been described as a “work-related syndrome” commonly characterized by high levels of emotional exhaustion, depersonalization, and dissatisfaction with one’s career and is associated with high turnover rates [10]. In this study, burnout is seen as an element within the broader concept of well-being when the negative impact on one’s well-being is at its extreme. The World Health Organization (WHO) projects a global needs-based shortage of health care workers at over 14.5 million in 2030 [11]. This workforce shortage has been deemed the “biggest, most pressing threat to the viability of services for people who need them” within the National Health Service (NHS) [12]. A bidirectional link between burnout and medical errors resulting in clinician distress has been observed, while conversely, better physician well-being was associated with improved patient satisfaction, improved treatment, and lower rates of hospital-acquired infections [13]. Thus, it is essential to find ways to improve well-being, for the benefit of the individual clinician, for patient care and safety, and to reduce provider costs [6].

Electronic health records (EHRs) are one example of an innovative tool that promised to empower clinicians; however, they have ended up being the focus of multiple studies of technologies’ negative impact on physician well-being [14,15]. The increased use of EHRs in health care organizations coincided with the need for prompt clinical data entry, and the lengthy and onerous clinical documentation process has been widely reported to have added to clinician frustration and, in many cases, been perceived to have added to administrative workload and time spent documenting rather than providing patient care [16,17]. Other dramatic changes in the ways clinicians work have been brought about with telemedicine [18], with studies also exploring the challenges of implementing AI, focusing on managers’ or users’ perspectives [19-21].

To date, research around burnout has mainly focused on doctors and nurses, with limited emphasis on other health care staff [22-24] and a holistic understanding of AI-enabled automation adoption. Some studies have shown several work system factors having a significant impact on predicting clinician burnout, including excessive workload, lack of job control through poor perceived autonomy and flexibility [11], misaligned values and expectations, lack of intrinsic motivation, and high administrative burden [9]. Other work system factors include workflow interruptions, poor technology usability that adds to the considerable workload and hinders workflow processes, moral distress, and time pressure. Recent research shows that health care staff perceive AI-driven tools to have the potential to help prevent burnout [25], and therefore, understanding the clinical environment and the impact of automation is crucial to improving the quality of care and patient safety.

The rising tide of AI-enabled digital health solutions places a growing emphasis on understanding the systemic impacts of implementing such technologies in health care and the subsequent impact on individuals. This study seeks to explore how staff members are impacted before and after the implementation of an autonomous clinical conversation agent in a routine elective care pathway.

### Intervention Used

This study will examine the impact on staff of the implementation of a UK Conformity Assessed-marked autonomous, voice-based, natural-language clinical assistant, “Dora” (Ufonia Ltd). Dora is simple and easy to use [3], as patients answer a telephone call they receive with no need for an internet connection, a smartphone, or a web interface. The technology can have a consultation with patients over the telephone, and thus the entire clinical conversation is tasked to Dora, which replaces the human conversation. For example, the key elements of a postoperative conversation in the cataract pathway with Dora are as follows:

- Greetings and introduction
- Confirmation of the identity of the patient
- Validated cataract follow-up questions
- Opportunity for the patient to ask Dora questions
- Decision regarding next steps of care
- Questions about acceptability (Net Promoter Score)
- Closure of the call

The technology has already demonstrated acceptability with patients in the cataract follow-up pathway, where it can reduce the number of patients needing a clinician-led consultation by up to 60% [3].

**Figure 1:**
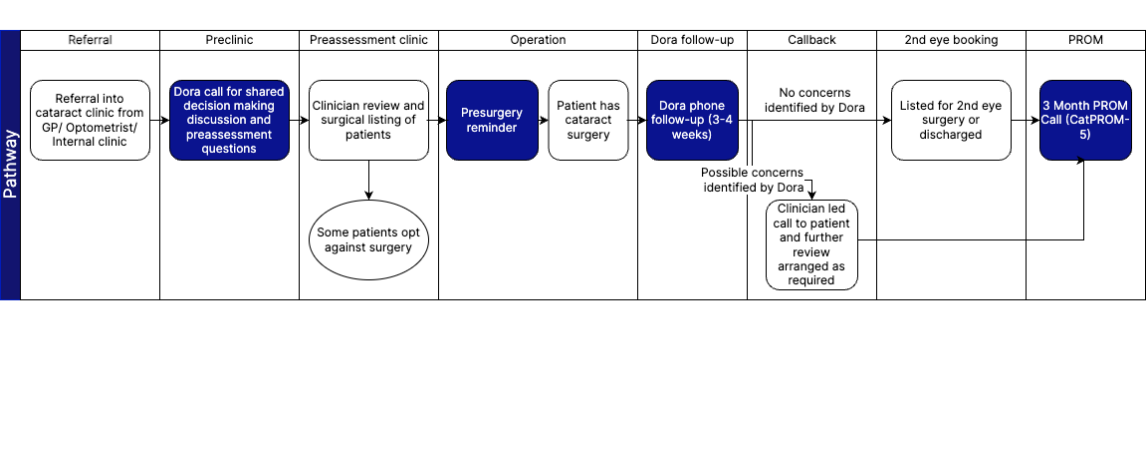
Example of an end-to-end Dora patient pathway for cataract surgery. PROM: patient-reported outcome measures.

Clinicians do not use any new interface. The department simply lists patients for a Dora call. Once Dora has conducted the clinical conversation, the results are fed back to the clinical team, who receive outcomes through a static report that summarizes the key domains assessed and the resulting outcome. This report is usually in the form of either a structured PDF file or a CSV file, depending on the required workflow. The Dora call can be implemented at multiple time points along the cataract pathway, as demonstrated in Figure 1. Apart from the patient pathway demonstrated in the figure below, this study will aim to shed light on the extent to which tasks and work practices are transferred or redesigned as a result of the introduction of Dora.

NHS South East (NHS-SE) has commissioned the regional use of Dora in the cataract pathway. Cataract surgery is the most common operation in the NHS, with over 450,000 procedures per year. Dora can have multiple conversations with patients, from preoperative assessment to postoperative review to assessing patient-reported outcome measures. The technology promises to automate these often stereotyped clinical interactions, potentially reducing the burden on clinicians multifold by replacing the conversation as well as automating the subsequent clinical documentation. In a pathway with Dora, rather than a clinician needing to consult every patient, only those with clinical concerns identified by Dora need a clinician review. This increases staff capacity while removing the stressors associated with repetitive routine conversations and administrative tasks such as documentation, targeting a major cause of clinician distress [26,27].

### Theoretical Framework

It is acknowledged that perception is a significant indicator of organizational readiness. Technology adoption theories, such as the Technology Adoption Model and diffusion of innovation theory, consider perception as a predictor of acceptance and use. These theories identified that previous experience, knowledge, and user skill sets influenced technology adoption in health care. For example, health care professionals willingly accepted the technology into their practice if it was aligned with professional values, was easy to use, trusted, and improved job performance [28,29].

While these perspectives could yield valuable insights, the sociotechnical system (STS) approach adds another dimension by acknowledging the interdependencies existing between organizations, individuals, and technology. This study adopts the STS perspective as a theoretical framework to analyze the impact of AI-enabled automation on the health care workforce and their work practices. The STS approach considers an organization as a work system with 2 interconnected subsystems: the technical dimension and the social dimension. The interactions between these 2 systems together produce the outputs of a work system, creating economic outcomes such as efficiency, productivity effectiveness, and cost reduction, in addition to humanistic outcomes such as well-being and engagement [30]. It is argued that performance is maximized only when the interdependence of these subsystems is explicitly recognized [31]. Sociotechnological studies on health care and technology implementation found that stakeholders hold different perspectives depending on their goals and expectations. For example, the organization’s goal of addressing workload productivity or maximizing financial performance may be different from a health care professional’s focus on improving patient outcomes and clinical decision-making. When introducing new technology into the health care industry, the challenge is determining the best method for comprehending the perceptions of all stakeholders. While the technical dimension of digital technologies and AI is currently dominating research studies, the impact on engagement, acceptance, and well-being of the individual is less well understood [25,30].

## Methods

### Study Design

To investigate the complex interactions between structures, people, organizations, technology, and contextually specific issues, a mixed methods research orientation will be adopted. This involves collecting, analyzing, and combining qualitative and quantitative data to obtain “complete analysis” [32,33] in understanding the relationship between technology and people and subsequent impact that cannot be fully understood by using a single methodology (ie, either qualitative or quantitative) [34,35]. Novel findings facilitate further investigation and expand the depth and scope of the data obtained. Furthermore, a mixed methods approach is suitable to answer both explanatory and confirmatory research questions.

The study will last for 12 months and consist of qualitative and quantitative data collection methods over 2 distinct phases, namely phase A (preimplementation) and phase B (postimplementation). Longitudinal data collection will assist in forming a richer understanding of the context, drawing further insights into how the change (ie, the intervention) impacts the workforce over time. The unit of analysis is the activity itself (the impact of AI-enabled automation on multiple stakeholders within the health care workforce), and within this focus of interest, tensions and contradictions will be identified and given particular priority and importance.

### Participant Recruitment

We will perform a multiple case study analysis as it favors the collection of rich data in multiple contexts, and the methodology allows for cross-case comparisons to clarify if the findings can be applied to several cases or are idiosyncratic to a single case. This will take place at multiple NHS hospital trusts. These sites represent a varied geographical location, baseline operational processes, management structure, demographics, and volume of patients. According to Benbasat et al [36], leveraging the case study approach is valuable for “problems... in which research and theory are at their early, formative stages and sticky, practice-based problems where the experiences of the actors are important and the context of action is critical.” Furthermore, multiple case study analysis enables the establishment of patterns or links between constructs within and across cases, together with their underlying logical explanations.

Participants will include doctors, nurses, allied health professionals, and administrators in the department where Dora is being implemented. Invitations to participate will be sent out through email and informed consent will be gained from participants before data collection. We intend to adopt a purposeful sampling technique, complemented by the snowballing technique, to identify knowledgeable informants. Participants will be chosen based on their role and knowledge of the study topic, as well as their involvement in the Dora pathway.

The final number of interviews will be determined while analyzing the data. For example, when no new codes and themes are emerging from the data and the analysis does not yield new themes but confirms current ones, it would suggest that a theoretical saturation point is obtained [37] and no new data are required.

### Data Collection

Multiple sources of evidence will be used, including semistructured interviews, focus groups, questionnaires, document analyses, and site visits, conducted by 2 researchers (SK and FZ). Using several forms of evidence enables the research team to understand the complexity of the context, obtain a rich data set, and facilitate triangulation, which improves the accuracy of the gathered data and enhances the credibility of the findings and conclusions [38]. Table 1 provides a summary of the methodology across the phases of intervention implementation.

**Table 1:** Summary table of methodology across phases of implementation.

Study and Dora implementation phases	Methodology	Description of workstream
**Phase A: preimplementation**		
	Structured survey	Distribute a survey to members of staff across multiple NHS^a^ sites to collect quantitative data before Dora implementation. Questions will focus on perceived acceptability, benefits, usability, and challenges associated with Dora.
	Interviews and focus groups	Conduct focus groups and semistructured interviews to understand the preconceptions that staff hold about automation using Dora and its potential impact on their work practices and well-being. Field notes will be taken during the interviews and consolidated after.
**Phase B: postimplementation**		
	Structured survey	Distribute the same survey as in phase A to enable comparisons between the initial data gathered before Dora implementation and after, highlighting any changes on an individual level.
	Interviews and focus groups	Conduct similar focus groups and interviews as in phase A to explore emergent themes in more depth and understand the postimplementation impact of Dora.

^a^NHS: National Health Service.

The questionnaire is designed to collect descriptive data about user acceptability, awareness, and well-being to aid in the interpretation of the findings (Table 2 provides an overview of the survey components). Before distributing the survey, it will first be piloted and validated by experts in the fields of health and information systems to ensure that it accurately measures health care professionals’ perceptions of the Dora intervention. The questionnaires will be complemented with focus groups and semistructured interviews (lasting between 30 and 60 minutes) to offset the lack of flexibility and depth of the survey and are developed to elicit critical process information that could add value to the survey data collected.

**Table 2:** Overview of questionnaire components and description.

Questionnaire component	Description
Demographics	Information about the hospital, job role, experience, work hours, age, gender, and ethnicity.
Dora involvement a understanding	Assesses the participant’s role in the Dora pathway and their self-rated knowledge of the system.
Stanford Professional Fulfillment and Burnout Index	Measures intrinsic positive reward derived from work and symptoms of work exhaustion and disengagement.
Impact of Dora on burnout	Evaluates the perceived impact of Dora on burnout symptoms.
Perceived acceptability	Gauges the acceptability of the Dora system, including user satisfaction and the system’s perceived value.
AI-related questions	Explores perceived benefits, performance anxiety, communication barriers, benefits, privacy concerns, liability, and risks.

Qualitative research methods involve the study of participants in their natural settings in order to interpret phenomena and the meanings individuals attribute to them. The fundamental goal is to understand participants from their own perspective [39]. To ensure trustworthiness, the study will be reported in accordance with the Consolidated Criteria for Reporting Qualitative Research (COREQ) 32-item checklist [40].

#### Preimplementation

A survey, based on the theoretical framework of acceptability and a validated well-being instrument (ie, Stanford Model of Professional Fulfillment), will be distributed to members of staff (both directly and indirectly affected by the intervention) across multiple NHS sites to collect quantitative data on well-being and perceptions before the implementation of Dora.

The aim of the survey is to gain an understanding of how AI-enabled automation, introduced by the intervention in this study, is perceived by the health care workforce. Questions will be based on perceived acceptability, benefits, and challenges associated with the implementation of Dora. This phase will also involve a focus group and semistructured interviews (conducted face-to-face or remotely, depending on convenience) to gain an understanding of the preconceptions held regarding automation using Dora, the impact of automation on themes such as autonomy, as well as the impact this may have on staff’s well-being. We will explore the system “as-is” to understand current ways of working, tensions and contradictions, and potential opportunities for Dora, to enable comparisons and further investigation. This will allow us to obtain baseline data to facilitate comparisons further into the study.

Focus groups enable participants to discuss this topic in greater depth and interact with, as well as react to, other participants [41], which can reveal shared opinions, reactions, and ideas on these phenomena. Field notes will be taken during the focus group and consolidated after.

The protocol used for the focus groups and interviews will be prepared based on the underlying constructs of the theoretical framework and literature review. These will provide a list of “intellectual bins” that structure the data collection and analysis [42]. Questions will largely be open-ended, giving participants the freedom to convey their personal views and experiences with respect to the socially complex context of AI implementation and automation in health care. The interviews will be conducted in a reflexive and responsive manner to enable the research team to follow up on insights and adjust the content of the interview accordingly. All interviews will be transcribed, annotated, and proofread. Furthermore, venting will be used, whereby interpretations and responses will be discussed with medical and academic professionals.

To enhance the robustness of the case studies, we will triangulate our findings by means of several evaluators (eg, researchers), multiple respondents, and different sources (ie, interviews, internal documentation).

#### Postimplementation

This phase involves the distribution of the same survey that was used in phase A as well as conducting a focus group and semistructured interviews with the same individuals contacted in phase A to explore emergent themes in more depth. This will enable comparisons between the initial data gathered before the implementation of Dora and the postimplementation, highlighting the changes that have occurred, if any, on an individual level.

### Data Analysis

The interview and focus group data will be thematically analyzed by the research team, which includes 2 researchers who are experienced in analyzing qualitative data. The first phase will involve the researchers familiarizing themselves with the data by reading and rereading the transcripts and field notes (also known as the “data immersion phase”). The raw data will be transferred to a qualitative analysis software (NVivo; QSR International) where it can be coded (ie, systematized and labeled). In this research, NVivo will facilitate the management and organization of data and the merging of codes, where appropriate, as part of the analysis.

A third researcher will further analyze a subset of the data to identify concepts that may have been missed, offer an alternative perspective, and detect possible bias in data analysis. We intend to adopt an insider and outsider approach to our coding and analysis to ensure rigor and help reduce bias. Coding will be regarded as complete when consensus is achieved on each construct, aiming to achieve a high intercode reliability score between the coauthors and the outsider researcher.

Guided by the research questions and theoretical lens, a set of initial codes will be applied to the data, forming the first-order codes. There is a possibility of creating additional codes, derived from open coding [43], to gain an in-depth understanding of the impact automation has on staff and their work practices, as framed by the participants (ie, in an inductive manner). This process will be followed by performing axial coding, where codes are categorized and organized into interpretive concepts. Finally, we will perform selective coding by aggregating the second-order codes into overarching theoretical constructs. The process will involve us cross-checking our analysis and looking for contradictions instead of seeking coherent and conformity interpretations [44].

During the data collection and analysis period, the research team will meet frequently to discuss the codes, review emerging themes, and develop and question each other’s ideas and underlying assumptions. The debriefings will enable us to develop a contextually specific shared understanding as well as determine a consolidated list of codes.

The quantitative data will be analyzed using quantitative analysis techniques to describe, explore, and examine the trends and relationships within the data. The results will be presented using charts and graphs to facilitate understanding.

### Data Handling

Before the interviews, participants will be contacted in writing to explain the study, and they will be asked to confirm their willingness to take part, which will be taken as consent to participate. There is no need for deception of any form in the research, and an information sheet outlining the research aim, purpose, and objectives, the right to withdraw, confidentiality assurances, and researchers’ contact details will be sent to all participants by email. With respect to the questionnaires, details about the study and a consent statement will be included at the start of the survey.

Data obtained from interviews will be anonymized and coded. Participants will be advised that any information given will be treated with strict confidence. All participants will be assured that their identity and names will not be included in later write-ups, work, or documents submitted or published.

Participants will be advised that they may stop being part of the study at any time without giving a reason, but information already collected by the research team will be kept.

## Results

The evaluation received funding from the MPS Foundation in December 2022, and the study will last 12 months. The expected results aim to reveal the interrelationship between the autonomous clinical conversational assistant and those affected by its introduction in the pathway, seeking to understand task redesign, changes in responsibilities, impacts on well-being, tensions, benefits, and opportunities.

The implementation of AI-enabled automation relies on the trustworthiness of the system, and any sense of insecurity may be stressful to the individual, impacting their own well-being and subsequently affecting system success. Employees’ attitudes and perceptions are argued to impact technology acceptance and attitudes toward change, which in turn affect the success of the intervention. Therefore, to achieve organizationally desired results from the adoption of AI-enabled automation technology, managers and leaders need to understand the opportunities and threats associated with its implementation and the impact this has on their workforce and their work practices.

The results from the thematic analysis of the qualitative data will be presented as the main themes that have been realized in the pre- and postimplementation phases. The data from the questionnaires will be presented in graphical format for each domain that has been assessed (Table 2 presents the questionnaire components).

## Discussion

Given the rising wave of AI-enabled digital health technologies, it has become paramount that the systemic impact of implementing such initiatives on individuals and the system overall be understood. AI advancements have created new possibilities for addressing a range of health care–related problems, with the promise of a positive impact on the working lives of frontline clinicians and other health care professionals. While most of the existing research on the impact of health information technology on well-being is concerned with clinicians’ EHR use, our current understanding of how AI impacts different members of staff and their roles and responsibilities remains opaque [25].

The primary aim of this study is to examine the real-world impact of AI-enabled automation on clinicians and health care professionals who provide health care to patients in high-volume, low-complexity perioperative care pathways. The results aim to shed light on concerns stakeholders have regarding automation and the use of telemedicine solutions, perceived benefits, and how the intervention impacts their work practices, if at all. The study will also highlight critical sociotechnical factors impacting adoption and whether there are any differences across different members of the health care workforce, which may allude to how managers can successfully integrate such technology into their organization and address the impact of such challenges. The study aims to bring original and novel contributions to the current body of knowledge as well as yield critical practical implications.

A few limitations have been identified. First, the study will be conducted within the specific research setting of the United Kingdom; therefore, some findings and interrelationships between them may be mainly applicable to contexts that are similar to the United Kingdom. Another limitation, and possibly an interesting direction of future research, is that although we aim to explore perceptions and experiences before and after implementation, there is opportunity to explore if there are any further changes over time and how these affect usage and deployment as the system becomes further embedded into the context.

## Abbreviations

AI: artificial intelligence
COREQ: Consolidated Criteria for Reporting Qualitative Research
EHR: electronic health record
NHS: National Health Service
NHS-SE: National Health Service–South East
STS: sociotechnical system
WHO: World Health Organization

## Data Availability

Data sharing is not applicable to this paper, as no data sets were analyzed for the creation of this study protocol.
